# Clinical and genetic variability in children with partial albinism

**DOI:** 10.1038/s41598-019-51768-8

**Published:** 2019-11-12

**Authors:** Patrick Campbell, Jamie M. Ellingford, Neil R. A. Parry, Tracy Fletcher, Simon C. Ramsden, Theodora Gale, Georgina Hall, Katherine Smith, Dalia Kasperaviciute, Ellen Thomas, I. Chris Lloyd, Sofia Douzgou, Jill Clayton-Smith, Susmito Biswas, Jane L. Ashworth, Graeme C. M. Black, Panagiotis I. Sergouniotis

**Affiliations:** 1 Manchester Centre for Genomic Medicine, Manchester Academic Health Sciences Centre, St Mary’s Hospital, Manchester University NHS Foundation Trust, Manchester, UK; 2 Division of Evolution and Genomic Sciences, Faculty of Biology, Medicine and Health, University of Manchester, Manchester, UK; 3 Manchester Royal Eye Hospital, Manchester Academic Health Science Centre, Manchester University NHS Foundation Trust, Manchester, UK; 4 Genomics England, London, UK; 5 Great Ormond Street Hospital for Children NHS Foundation Trust, London, UK

**Keywords:** Paediatric research, Genetics research, Molecular medicine, Disease genetics, Eye manifestations

## Abstract

Individuals who have ocular features of albinism and skin pigmentation in keeping with their familial background present a considerable diagnostic challenge. Timely diagnosis through genomic testing can help avert diagnostic odysseys and facilitates accurate genetic counselling and tailored specialist management. Here, we report the clinical and gene panel testing findings in 12 children with presumed ocular albinism. A definitive molecular diagnosis was made in 8/12 probands (67%) and a possible molecular diagnosis was identified in a further 3/12 probands (25%). *TYR* was the most commonly mutated gene in this cohort (75% of patients, 9/12). A disease-causing *TYR* haplotype comprised of two common, functional polymorphisms, *TYR* c.[575 C > A;1205 G > A] p.[(Ser192Tyr);(Arg402Gln)], was found to be particularly prevalent. One participant had *GPR143*-associated X-linked ocular albinism and another proband had biallelic variants in *SLC38A8*, a glutamine transporter gene associated with foveal hypoplasia and optic nerve misrouting without pigmentation defects. Intriguingly, 2/12 individuals had a single, rare, likely pathogenic variant in each of *TYR* and *OCA2* – a significant enrichment compared to a control cohort of 4046 individuals from the 100,000 genomes project pilot dataset. Overall, our findings highlight that panel-based genetic testing is a clinically useful test with a high diagnostic yield in children with partial/ocular albinism.

## Introduction

Albinism is a group of hereditary conditions characterised by decreased or absent ocular pigmentation and variable skin/hair pigmentation. It can be broadly subdivided into oculocutaneous albinism (OCA) and ocular albinism (OA) [MIM: 300500]. Syndromic forms of albinism have also been described (including Hermansky-Pudlak [MIM: 203300] and Chediak-Higashi syndromes [MIM: 214500]); in these rare disorders, pigmentation abnormalities co-exist with other pathological alterations^1^. Ocular features are present in all albinism patients and are characteristic of the condition; these may include nystagmus, foveal hypoplasia, fundal hypopigmentation, iris transillumination and optic nerve misrouting. In contrast, cutaneous features may or may not be present^2^.

Albinism, and OCA in particular, exhibits significant clinical and genetic heterogeneity^3,4^. Notably, skin pigmentation abnormalities can often be difficult to evaluate, especially in people with light-skinned familial backgrounds. In such cases, there is significant phenotypic overlap with a range of other ophthalmic conditions including X-linked idiopathic congenital nystagmus [MIM: 310700]^5^, SLC38A8-associated foveal hypoplasia (also known as FHONDA [MIM: 609218], a condition including optic nerve decussation defects and, in some cases, anterior segment dysgenesis)^6^, and dominant *PAX6*-related oculopathy [MIM: 136520]^7^. Obtaining a precise diagnosis and distinguishing between these different entities is particularly important as it enables accurate genetic counselling and personally tailored surveillance and management decisions (e.g. dermatological input). However, the current diagnostic pathway for these individuals is often prolonged, cumbersome and frequently unsuccessful. Investigations such as slit lamp examination, macular optical coherence tomography and visual electrophysiology can be helpful and are typically used to formally diagnose iris transillumination, foveal hypoplasia and optic nerve misrouting respectively. However, performing such tests in infants and young children can be challenging as co-operation for several minutes at a time is required. Furthermore, the ocular signs of partial albinism can be subtle and certain features may be absent in mild cases^8^. As a result, multiple clinic visits over years and several investigations may be needed to reach a formal diagnosis.

The use of genetic testing early in the care pathway offers significant potential for accelerating diagnosis in individuals suspected to have partial OCA. Over the past decade, advances in DNA sequencing technologies including gene panel testing and genome sequencing have revolutionised diagnostics across many heterogeneous inherited disorders^9^. A recent report on the use of a panel-based sequencing approach in a large cohort of patients with albinism found that a definitive molecular diagnosis could be obtained in 72% of cases^10^. A notable finding of this study was the confirmation of a *TYR* allele containing two relatively common variants *in cis TYR* c.[575 C > A;1205 G > A] p.[(Ser192Tyr);(Arg402Gln)] as a prevalent cause of mild albinism when *in trans* with a *TYR* pathogenic variant^3,10,11^. Despite this recent progress in dissecting the molecular pathology of albinism, our understanding of the genetic basis of partial OCA remains incomplete.

Here, we studied 12 individuals who presented diagnostic challenges as they had some ocular features of albinism but their skin was not hypopigmented in the context of their family. Phenotypic and gene panel testing results are discussed. The clinical utility of early genomic testing in this group of patients is highlighted.

## Results

### Clinical findings

We identified 12 children (under 16 years of age at time of testing) with nystagmus, at least one other ocular feature of albinism, and no apparent skin hypopigmentation in the context of their family. Their clinical features are summarised in Table 1. Nystagmus was the presenting symptom in all patients and the median age at referral for genetic testing was 5.5 years. Foveal hypoplasia was suspected in 11/12 and iris transillumination was detected in 7/12. Possible electrophysiological evidence of optic nerve misrouting was identified at some point in the life of 9/10 patients tested; 3 of these 9 children were tested more than once and there was variability in the visual evoked potential (VEP) findings between visits (Table 1). For example, proband 2 had electrodiagnostic studies at age 9 months which showed minimal crossed asymmetry. When the test was repeated at age 2 years, there was no evidence of crossed asymmetry but a further assessment at age 14 years showed marked crossed asymmetry. This is perhaps not surprising as the visual system undergoes dramatic maturationial change in the first few years of life which is manifested in flash and pattern VEPs^12,13^, leading to the recommendation to wait until 4 years of age before repeating ambiguous VEPs.

**Table 1:** Clinical findings in 12 children with ocular features of albinism and no obvious skin pigmentation abnormalities.

proband ID	age at genetic testing (years)	sex	iris transillumination	foveal hypoplasia	fundal hypopigmentation	age at VEP testing (years)	VEP crossed asymmetry (pattern & flash VEP score)	relevant family history
1	<1	M	no	yes	yes	<1	possible (P-1, F1)	none reported
2	14	F	no	yes	yes	<1; 2; 14	probable (P-1, F2); no (P-1, F0); yes (P3, F3)	none reported
3	1	M	yes	yes	yes	not tested	not tested	none reported
4	3	M	yes	yes	yes	<1	yes (P3, F3)	mother with iris transillumination & foveal hypoplasia
5	6	M	yes	yes	yes	5	yes (P3, F3)	identical twin affected
6	1	M	yes	yes	yes	<1	yes (P3, F3)	none reported
7	8	M	yes	yes	yes	<1	probable (P2, F1)	none reported
8	11	F	yes	yes	yes	not tested	not tested	brother affected
9	1	F	yes	yes	yes	1	no (P0, F0)	none reported
10	7	F	no	yes	yes	3; 6	probable (P2, F0); probable (P0, F2)	none reported
11	7	M	no	yes	no	1; 4	possible (P1, F0); no (P0, F0)	half-brother of paternal grandmother with nystagmus
12	5	M	no	no	no	2	yes (P1, F3)	parental consanguinity

VEP, visual evoked potential; M, male; F, female. All study participants had skin pigmentation in keeping with their familial background. Other clinical features included prominent posterior embryotoxa in proband 2, easy bruising in proband 9 and mild developmental delay in proband 12.

Pattern (P) and flash (F) VEP scores are shown. A score of [−1] corresponds to inadequate signal, either due to poor cooperation or simply because spatial vision was too poor. A score of [0] suggests no crossed asymmetry (i.e. the right-left difference plots for right and left eyes were indistinguishable). A score of [1] denotes possible crossed asymmetry (i.e. whilst most components are not asymmetrical, one or two are). A score of [2] suggests probable crossed asymmetry (i.e. crossed asymmetry is partial or the polarity of the components is reversed but the phase is shifted). A score of [3] denotes definite crossed asymmetry (i.e. while right-left interhemispheric difference plots may be of differing amplitude, their waveforms are more or less mirror images of each other.

### Genetic findings and diagnostic pick-up rate

A probable molecular diagnosis was identified in 8/12 patients (67%); a possible molecular diagnosis was identified in a further 3/12 patients (25%). Overall, 10/12 children were found to have pathogenic variants in OCA or OA-associated genes. The most frequently implicated gene was *TYR*: sequence alterations in this gene were identified in 75% of patients (9/12). One child was found to have a hemizygous pathogenic variant in *GPR143* [MIM: 300808], the gene associated with X-linked OA, and another child was found to have a compound heterozygous changes in *SLC38A8*, a gene associated with foveal hypoplasia and optic nerve misrouting without pigmentation abnormalities. We identified four previously unreported disease-causing changes: two in *SLC38A8*, one in *TYR* and one in *OCA2*. The genetic findings in all study participants can be found in Table 2 and further details on the individual variants are in Table 3. Familial segregation is discussed below and family trees are provided in the Supplementary File.

**Table 2:** Genetic findings in 12 children with ocular features of albinism and no obvious skin pigmentation abnormalities.

proband ID	gene panel used for testing (number of genes evaluated)	variant 1	variant 2	variant 3	samples available for segregation
1	ocular/oculocutanous albinism (18)	*GPR143* c.659-1 G > A	—	—	mother
2	nystagmus & foveal hypoplasia (26)	*SLC38A8* c.534 C > G p.(Ile178Met)	SLC38A8 exon 2 to 5 deletion	—	none
3	ocular/oculocutanous albinism (18)	*TYR* c.1217 C > T p.(Pro406Leu)	*TYR* c.575 C > A p.(Ser192Tyr)	*TYR* 1205 G > A p.(Arg402Gln)	none
4	optic nerve disorders (40)	*TYR* c.1118 C > A p.(Thr373Lys)	*TYR* c.575 C > A p.(Ser192Tyr)	*TYR* 1205 G > A p.(Arg402Gln)	mother & father
5	ocular/oculocutanous albinism (18)	*TYR* c.823 G > T p.(Val275Phe)	*TYR* c.[575 C > A; 1205 G > A] p.[(Ser192Tyr);(Arg402Gln)]	*OCA2* c.1327 G > A p.(Val443Ile)	mother & father
6	nystagmus & foveal hypoplasia (26)	*TYR* c.1118 C > A p.(Thr373Lys)	*TYR* c.[575 C > A; 1205 G > A] p.[(Ser192Tyr);(Arg402Gln)]	—	mother & father
7	nystagmus & foveal hypoplasia (26)	*TYR* c.[575 C > A; 1205 G > A] p.[(Ser192Tyr);(Arg402Gln)]	*TYR* c.[575 C > A; 1205 G > A] p.[(Ser192Tyr);(Arg402Gln)]	—	no
8	ocular/oculocutanous albinism (18)	*TYR* c.[575 C > A; 1205 G > A] p.[(Ser192Tyr);(Arg402Gln)]	*TYR* c.[575 C > A; 1205 G > A] p.[(Ser192Tyr);(Arg402Gln)]	*TYRP1* c.208 G > A p.(Ala70Thr)	mother, father & affected brother
9	clinical exome	*OCA2* c.1327 G > A p.(Val443Ile)	*TYR* c.575 C > A p.(Ser192Tyr)	*TYR* 1205 G > A p.(Arg402Gln)	no
10	nystagmus & foveal hypoplasia (26)	*OCA2* c.2346deIG p.(Thr783Hisfs*2)	*TYR* c.1392dupT p.(Lys465*)	—	mother
11	nystagmus & foveal hypoplasia (26)	*OCA2* c.1441 G > A, (p.Ala481Thr)	*TYR* c.1217 C > T, p.(Pro406Leu)	—	mother
12	ocular/oculocutanous albinism (18)	no pathogenic variant identified	—	—	no

The genes and transcripts included in each gene panel can be found in Supplementary Table 1. The results of segregation are discussed in the text and family trees are provided in the Supplementary File.

**Table 3:** Analysis of disease-associated variants identified in children with ocular features of albinism.

gene	genotype	protein	probands affected	report describing the variant in association with albinism	gnomAD total frequency % (allele count)	polyphen-2 HumVar score	CADD score
*GPR143*	c.659-1 G > A	not applicable	1	Han *et al*.^15^	not detected (0/183,366)	not applicable	26.5
*TYR*	c.1217 C > T	p.(Pro406Leu)	3,11	Giebel *et al*.^18^	0.3918% (1,104/281,766)	0.997	27.2
*TYR*	c.[575 C > A; 1205 G > A]	p.[(Ser192Tyr);(Arg402Gln)]	3, 4, 5, 6, 7, 8, 9	Norman *et al*.^3^	25.02% (70,744/282,804) & 17.65% (49,703/281,606)	0.974 & 0.994	24.2 & 29.4
*TYR*	c.1118 C > A	p.(Thr373Lys)	4, 6	King *et al*.^19^	0.0354% (100/282,382)	0.004	23.5
*TYR*	c.823 G > T	p.(Val275Phe)	5	Giebel *et al*.^18^	0.0099% (28/282,378)	0.42	11.17
*TYR*	c.1392dupT	p.(Lys465*)	10	novel	not detected (0/251,158)	not applicable	not applicable
*SLC38A8*	c.534 C > G	p.(Ile178Met)	2	novel	0.0016% (4/250,998)	0.701	22.1
*SLC38A8*	exon 2 to exon 5 deletion	exon 2 to exon 5 deletion	2	novel	not applicable	not applicable	not applicable
*OCA2*	c.1327 G > A	p.(Val443Ile)	5, 9	Lee *et al*.^20^	0.3055% (860/281,442)	0.998	27.0
*OCA2*	c.2346deIG	p.(Thr783Hisfs*2)	10	novel	not detected (0/251,472)	not applicable	not applicable
*OCA2*	c.1441 G > A,	p.(Ala481Thr)	11	Yuasa *et al*.^22^	0.8427% (2,384/282,886)	0.466	24.7
*TYRP1*	c.208 G > A	p.(Ala70Thr)	8	Marti *et al*.^21^	0.0242% (68/281,344)	0.606	23.0

Polyphen-2^22^ (http://genetics.bwh.harvard.edu/pph2/) predicts the impact of an amino acid substitution on a human protein using physical and comparative considerations. The output is a 0 to 1 score; the higher the score the more likely it is that the variant is pathogenic. Combined Annotation-Dependent Depletion^23^ (CADD; https://cadd.gs.washington.edu/) combines information from many different in silico tools and uses a support vector machine classifier. The CADD score ranges from 1 to 99; a higher score indicates greater pathogenicity. Values ≥10 are predicted to be the 10% most deleterious substitutions and ≥ 20 in the 1% most deleterious. The overall gnomAD^26^ (http://gnomad.broadinstitute.org/) minor allele frequency is presented. Polyphen-2, CADD and gnoMAD were all accessed on 23/01/2019.

#### Proband 1

Proband 1, a 1-year-old white British male, was found to have the *GPR143* c.659-1 G > A change in hemizygous state. This variant was not detected in a maternal sample and is not present in gnomAD^14^. It has, however, previously been reported in a Chinese male patient with OA^15^.

#### Proband 2

**Figure 1:**
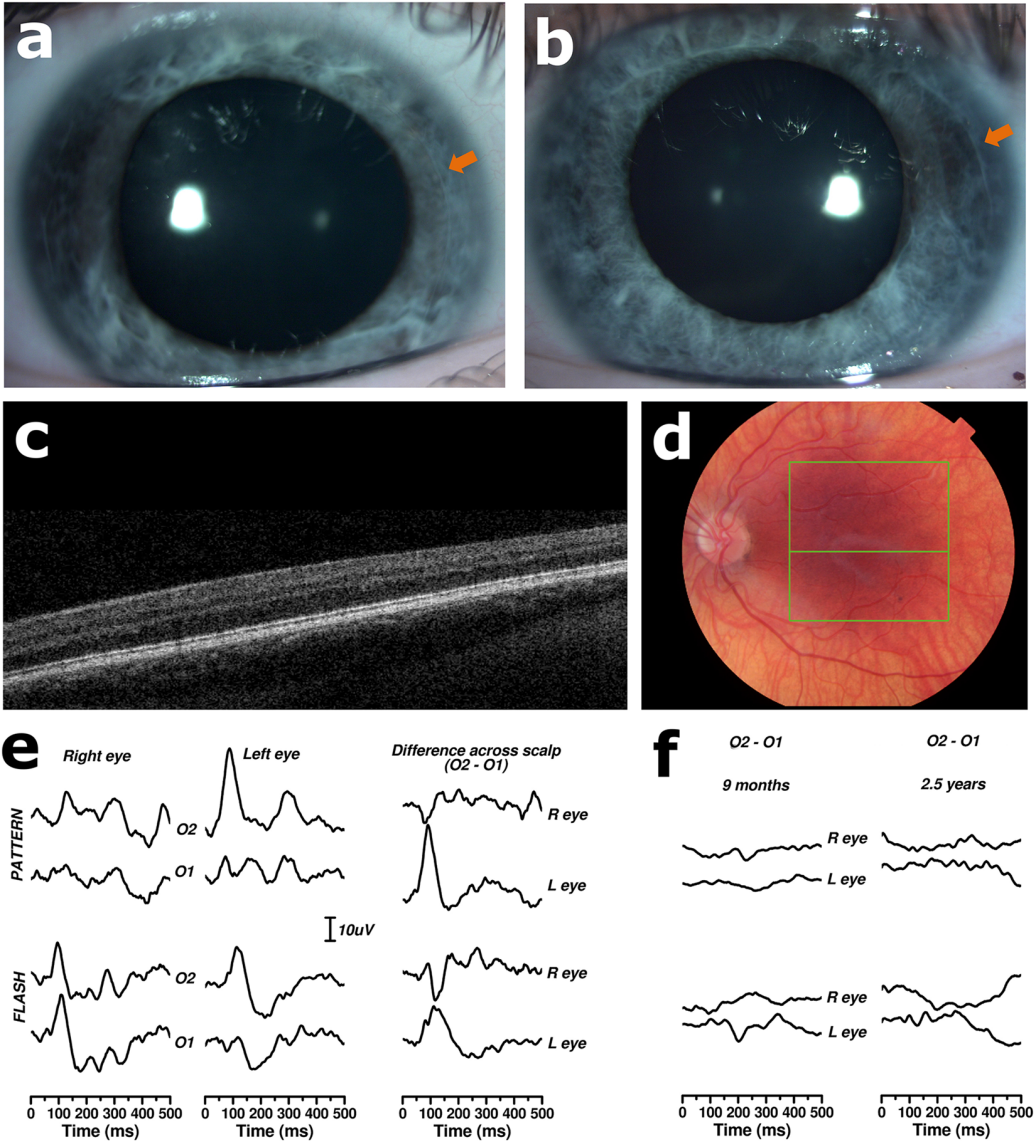
Ocular and electrodiagnostic findings in a patient with *SLC38A8*-associated disease (proband 2). (**a**,**b**) Anterior segment photographs of right (**a**) and left (**b**) eyes obtained at age 14 years. The arrows mark the location of the embryotoxa. (**c**,**d**) Linear optical coherence tomography (OCT) scan through the centre of the macula of the left eye demonstrating absence of the foveal depression in keeping with foveal hypoplasia. (**c**) The associated left colour fundus photograph highlighting the location of the scan (line in the middle of the green box) is also shown. This image (**d**) reveals that, although the central macula appears to be adequately pigmented, there is a degree of fundal hypopigmentation mid-peripherally. It is noteworthy that the proband is of white British background and her skin was not hypopigmented in the context of her family. These images were obtained when the patient was 12 years of age. (**e**) Monocular pattern and flash visual evoked potentials (VEPs) recorded from electrodes mounted 3 cm to right (O2) and left (O1) of midline. The patient was 14 years old at the time of testing. The difference plots are subtractions of left from right scalp recordings to show how scalp asymmetry is reversed in the two eyes (crossed asymmetry) when virtually all optic nerve fibres cross at the chiasm. (**f**) Difference plots recorded at age 9 months and 2.5 years, showing minimal or no crossed asymmetry; this is in contrast to the assessment at age 14 years, shown in (**e**), which was in keeping with marked crossed asymmetry.

Proband 2, a 14-year-old white British female, was found to have compound heterozygous changes in the *SLC38A8* gene; these included a heterozygous missense change c.534 C > G p.(Ile178Met) and a heterozygous large inframe deletion removing exons 2 to 5. The missense mutation has not been previously reported as disease causing. It is found at very low frequency in population databases and it is reported as possibly damaging by *in silico* tools (Table 3). The large deletion removes 167 amino acids (the total length of the protein is 435 amino acids) affecting 5 of the 11 transmembrane domains of this glutamine transporter. Notably, the patient has prominent posterior embryotoxa (Fig. 1). Such abnormalities have been described in 3 of the 9 families with *SLC38A8*-associated disease reported to date^6,16,17^.

#### Probands 3, 4, 5 and 6

**Figure 2:**
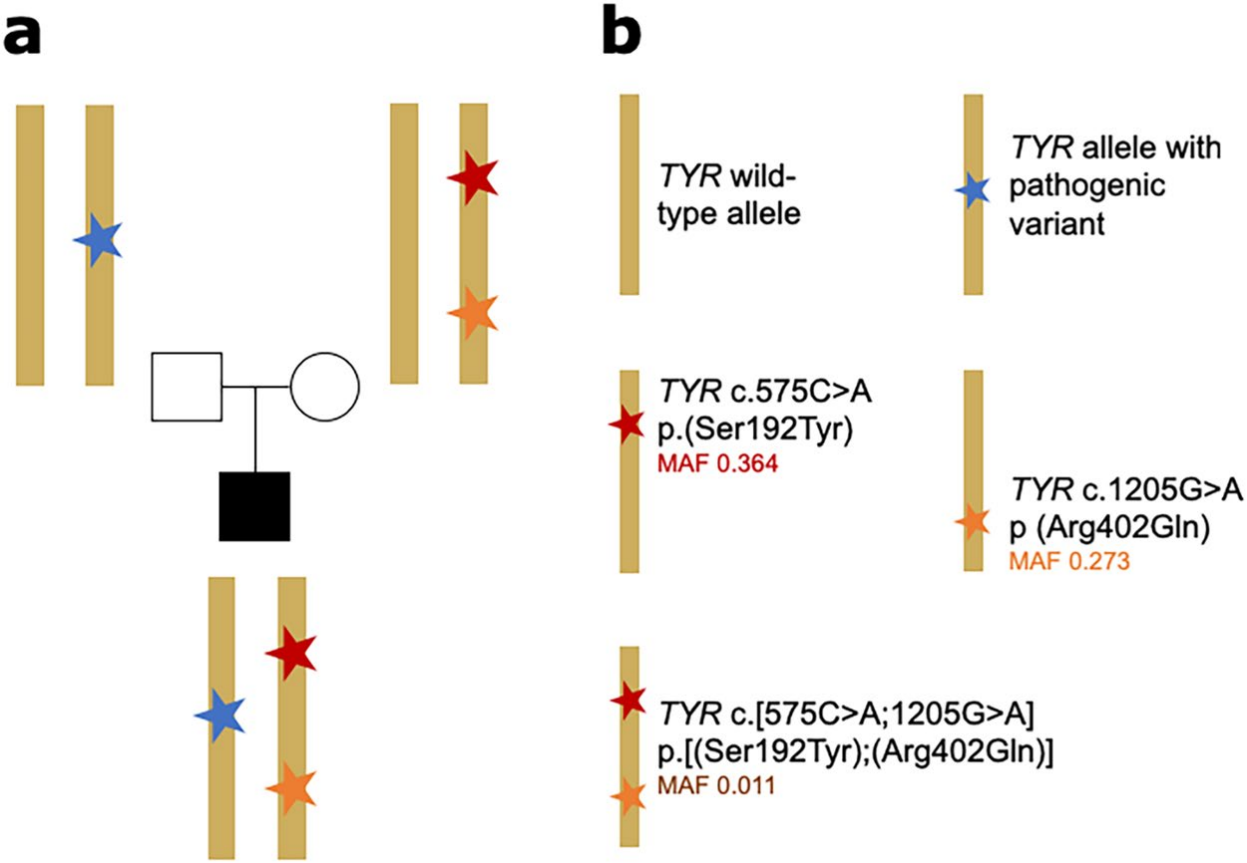
Schematic representation of the formation of the *TYR* c.[575 C > A;1205 G > A] p.[(Ser192Tyr);(Arg402Gln)] complex allele. (**a**) Basic pedigree including parental haplotypes from an individual (proband 6) carrying the TYR complex allele (red & orange stars) *in trans* with a TYR pathogenic allele (blue star). (**b**) Figure key for (**a**) explaining the variants present in each allele. Notably, each of c.575 C > A p.(Ser192Tyr) and c.1205 G > A p (Arg402Gln) has been shown to predispose to decreased pigmentation^40,41^ and to result in reduced tyrosinase activity^11^. The minor allele frequencies (MAF) shown for these two variants are from the gnomAD variant database^25^ (accessed 23/01/2019, European non-Finnish population). The MAF shown for the c.[575 C > A;1205 G > A] p.[(Ser192Tyr);(Arg402Gln)] complex allele is estimated using data from the “British in England & Scotland” subset of the 1000 genomes project^3^.

In four probands (3, 4, 5 and 6), we identified a known disease-associated *TYR* variant along with a genotype in keeping with the presence of the *TYR* complex haplotype p.[(Ser192Tyr);(Arg402Gln)] (Table 2; Fig. 2). It was possible to perform segregation analysis only in probands 4, 5 and 6 (please see Supplementary File for more details). For proband 4 it was not possible to verify *cis/trans* phase of the variants. In proband 5 and 6, the *TYR* variants p.(Arg402Gln) and p.(Ser192Tyr) were found to be *in cis* and the resultant haplotype – p.[(Ser192Tyr);(Arg402Gln)] – was verified as being *in trans* with known pathogenic *TYR* variants: *TYR* c.823 G > T p.(Val275Phe)^18^ in proband 5 and *TYR* c.1118 C > A p.Thr373Lys^19^ in proband 6 (Fig. 2). An established pathogenic variant in *OCA2* – *OCA2* c.1327 G > A p.(Val443Ile)^20^ – was also identified in proband 5.

#### Probands 7 and 8

In probands 7 and 8, both the *TYR* p.(Arg402Gln) and p.(Ser192Tyr) variants were detected in a homozygous state. These two probands had all the cardinal ocular features of albinism but their skin pigmentation was in keeping with their wider family. Proband 8 was also found to have a rare *TYRP1* variant: *TYRP1* c.208 G > A p.(Ala70Thr). This variant is reported to be possibly damaging by *in silico* tools (Table 3) and a recent study identified the variant in a patient with albinism^21^. Proband 8 also has a 1-year-old brother with nystagmus who was also homozygous for *TYR* p.(Arg402Gln) and *TYR* p.(Ser192Tyr) but did not carry the *TYRP1* p.(Ala70Thr) variant.

#### Probands 9, 10 and 11

Probands 9, 10 and 11 were found to have heterozygous disease-causing variants in each of the *TYR* and *OCA2 genes*. Proband 9 had a single, previously reported disease-associated variant in *OCA2* – *OCA2* p.(Val443Ile)^20^ – and is also heterozygous for the *TYR* c.575 C > A p.(Ser192Tyr) and *TYR* c.1205 G > A p.(Arg402Gln) variants; segregation analysis was not possible. Proband 10 had two previously unreported heterozygous truncating variants, one in *TYR* c.1392dupT p.(Lys465*) and one in *OCA2* c.2346deIG p.(Thr783Hisfs*2). Her unaffected mother was found to only carry the *TYR* variant. Proband 11 harboured a previously reported disease-associated variant in *TYR* – *TYR* c.1217 C > T p.(Pro406Leu)^18^ – and a hypomorphic allele in *OCA2* – *OCA2* p.(Ala481Thr)^22^. This *OCA2* change has previously been suspected to cause albinism in some patients but reports on ClinVar are conflicting^23,24^. A maternal sample was available for segregation revealing that the proband’s mother carried both the *TYR* and *OCA2* alleles but in the absence of a clinical phenotype of partial OCA. This result raises questions over the penetrance/pathogenicity of the genotype in proband 11. However, according to the criteria outlined in the methods section, proband 11 meets the definition for having a possible molecular diagnosis.

#### Proband 12

In proband 12, no pathogenic variants were identified.

### Prevalence of selected genotypes in an external cohort

To gain insights into the prevalence of certain combinations of variants in individuals without a clinical diagnosis of albinism, we interrogated the genomic data of 4046 individuals from the 100,000 genomes project pilot dataset^25^. First, we looked at how many individuals in this external dataset were homozygous for the *TYR* p.[(Ser192Tyr);(Arg402Gln)] haplotype (like probands 7 & 8); two of 4046 individuals were found to be homozygous for both *TYR* p.(Arg402Gln) and p.(Ser192Tyr). Then, we looked for individuals that have a genotype in keeping with the presence of the *TYR* p.[(Ser192Tyr);(Arg402Gln)] haplotype in combination with a rare (MAF < 0.047 in ExAC^14^) *TYR* variant (like probands 3–6). Six of the 4046 individuals in the external cohort met this criterion. In all these six cases data from other family members were available; we therefore assessed the phase of the variants in the complex *TYR* allele and found that in all these individuals p.(Arg402Gln) and p.(Ser192Tyr) were highly likely to occur *in trans*. Finally, we looked for individuals that had both a rare *TYR* variant and a rare *OCA2* variant (like probands 10 & 11). One of the 4046 individuals in the external cohort met this criterion. Evaluation using the Fisher exact test revealed that each of these 3 variant combinations is significantly enriched in the patient cohort compared to the external, 100,000 genomes project pilot cohort (p-value of < 0.0001 for each of the 3 comparisons).

## Discussion

Although the study of severe, complete albinism phenotypes is relatively straightforward, milder subtypes remain difficult to diagnose clinically. Recent studies have made considerable progress in appreciating the clinical heterogeneity and elucidating the genetic basis in this group of conditions^3,8,10,26^. Here, we identified a cohort of children who have ocular features of albinism and skin pigmentation that is in keeping with their familial background. Panel-based genetic testing was performed and the current utility of this investigation in clinical practice was evaluated

Detailed phenotyping of study participants highlighted the heterogeneity of partial albinism. There were varying combinations of ophthalmic symptoms and signs present in each child. This observation is in line with a recent study on the phenotypic spectrum of albinism that found no pathognomonic features for this condition^8^. This study by Kruijt and colleagues^8^ represents a significant milestone towards establishing clinical criteria for albinism. However, although the described classification system is precise and carefully designed, it is also dependent upon high-quality phenotypic information (such as OCT-based grading of foveal hypoplasia) that can be challenging to routinely and consistently obtain in infants and young children. Given this issue and the significant clinical variability, genetic testing represents an important method for obtaining a precise, timely diagnosis in children with partial albinism. We utilised a panel test for genes known to cause albinism or plausible differential diagnoses which in all cases contained 18 common genes linked to albinism (Supplementary Table 1). We established a high diagnostic rate with 92% (11/12) of study participants receiving a probable or possible molecular diagnosis. This finding is comparable to that of a recent report on albinism that identified biallelic or monoallelic variants in relevant genes in 85% (837/990) of patients with albinism^10^. We found that most individuals in the present partial albinism cohort (10/12) had mutations in OA/OCA-related genes. *TYR* was the most frequently mutated gene: 6/8 (75%) patients who received probable molecular diagnosis had TYR-associated disease. In contrast, Lasseaux and colleagues, in a larger and more heterogeneous albinism cohort (including individuals with both complete and incomplete forms), reported identifying *TYR* mutations in 300/717 (42%) of the solved cases s^10^.

Notably, one patient in in the present study had *SLC38A8*-associated disease, an important differential diagnosis of partial albinism. *SLC38A8* encodes a glutamate transporter that, unlike OA/OCA-related genes, has not been linked with defects in melanin biosynthesis or melanocyte differentiation^6^. Individuals with this condition present with nystagmus, foveal hypoplasia and optic nerve misrouting without iris, fundal or skin pigmentation defects (Fig. 1)^6,16,17^. Prominent posterior embryotoxa are present in some cases (including proband 2) and the presence of this anterior segment sign in an individual with partial albinism should raise suspicion for *SLC38A8*-associated disease.

In this cohort, *TYR* c.[575 C > A;1205 G > A] p.[(Ser192Tyr);(Arg402Gln)] was the most common pathogenic allele as it was suspected to be present in 7/12 patients. The two genotypes *TYR* c.575 C > A and *TYR* c.1205 G > A have arisen on different ancestral haplotypes^27^ and each is known to reduce pigmentation^28,29^. Individually both variants are common (gnomAD frequencies: 25.02% and 17.65% respectively) but their presence together on a recombinant haplotype *TYR* c.[575 C > A;1205 G > A] p.[(Ser192Tyr);(Arg402Gln)] is a relatively rare event, predicted to be found in British patients at a frequency of 1.1%^3^. This haplotype has been found to lead to an additive decrease in tyrosinase function compared to each of the two variants individually (Fig. 2)^11^. Recent genome sequencing in nine individuals with albinism carrying this haplotye and follow-up functional work did not support any other variants on the haplotype as having a role in reducing TYR function^26^. It appears likely that this haplotype is pathogenic because the combination of these two functional coding variants reduces TYR production to pathogenic levels. This recombination of common single polymorphisms that aberrantly affect protein function onto a single haplotype, with resulting significant decrease in protein function (in an additive or multiplicative fashion) to pathogenic levels is a rarely reported mechanism. Although initially described as a ‘tri-allelic genotype’ by Norman *et al*. (2017), we feel it would be better to classify this as a ‘complex’ or ‘pathogenic’ haplotype, like Grønskov *et al*.^26^, to reflect these mechanistic underpinnings. Such a ‘complex/pathogenic’ haplotype has also recently been noted in *ABCA4-*related eye disease^30^. The discovery of such alleles re-enforces the importance of considering *cis-*genomic context in mutational analysis^31^. Current genetic laboratory pipelines are often suboptimally set up to identify these complex disease-causing haplotypes as data are filtered based on the rarity of individual variants rather than haplotypes. However, complex/pathogenic alleles may underlie some of the current missing heritability in rare genetic disease. Detailed analysis of individuals with monoallelic pathogenic variants in genes associated with recessive disorders offers the best chance to identify such haplotypes, as has happened serendipitously in albinism.

We identified two individuals with partial albinism that were homozygous for the *TYR* p.[(Ser192Tyr);(Arg402Gln)] allele. Compared to a control cohort from the 100,000 genomes project pilot dataset, this genotype is significantly enriched among albinism patients. Grønskov *et al*.^26^ identified six further individuals with mild albinism that were homozygous for the haplotype in their cohort of 93 albinism patients; five of these individuals were clinically diagnosed with OA – supporting the mild cutaneous phenotype associated with this genotype. Grønskov *et al*.^26^ also noted that, given the high predicted carrier frequency of this haplotype in the general population, (i) it would be surprising if it were a fully penetrant disease-associated allele and (ii) mild cases of albinism may remain undiagnosed/asymptomatic especially in light-skinned population. Indeed, homozygosity for the complex haplotype may only cause ocular de-pigmentation that is significant enough to prompt tertiary referral for ophthalmological complications upon an already low pigmentation background.

We identified two individuals with partial albinism and monoallelic rare variants in both *TYR* and *OCA2* – a significant enrichment compared to a control cohort from the 100,000 genomes project pilot dataset. We with “considered these individuals as having a possible molecular diagnosis given that they have a variant in a gene linked with recessive disease and a strong genotype-phenotype fit. Intriguingly, oligo/digenic inheritance mechanisms have been hypothesised previously in albinism^32–34^ and given that melanin biosynthesis is known to be an interactive pathway this remains an attractive hypothesis^35,36^. However, convincing evidence is yet to be reported in the scientific literature.

This study has a number of limitations. A notable one relates to the fact that the panel tests utilised here did not include three genes previously linked to rare forms of ‘syndromic albinism’, *HSP1*^37^, *AP3D1*^38^, *MITF*^39^, and one gene recently linked to ocular albinism, *GNAI3*^40^. In addition to being unable to rule out defects in these four genes, we did not evaluate non-coding variation associated with known albinism-related genes. Further studies of probands and families with an eluding molecular diagnosis would be of interest to identify the sources of the remaining missing heritability in albinism.

In conclusion, we report that panel-based genetic testing has a high diagnostic rate in ocular/partial albinism suspects. It has the potential to allow timely diagnosis whilst avoiding multiple clinical assessments and diagnostic tests. Also, it impacts clinical management; for example, individuals with confirmed OCA will receive appropriate support and advice regarding skin care. Notably, variants in OCA-related genes account for most genetic diagnoses in this cohort of patients. Findings in a small but significant minority of individuals with partial albinism suggest that investigating the penetrance of *TYR* variants and exploring a digenic inheritance model involving *TYR* and *OCA2* would be of interest. However, large, collaborative research cohorts with high quality phenotyping and familial segregation data would be required to unravel these genetic phenomena.

## Methods

### Editorial policies and ethical considerations

This study obtained ethics approval from the North West Research Ethics Committee (11/NW/0421 and 15/YH/0365). Informed consent was gained from all patients (or their respective parental guardians) prior to ophthalmic examination and genetic testing. The research adhered to the tenets of the Declaration of Helsinki.

### Patient ascertainment and phenotypic data collection

Children (under 16 years of age) with: (i) nystagmus, (ii) at least one other ocular feature of albinism (iris transillumination, foveal hypoplasia, optic nerve misrouting) and (iii) skin pigmentation in keeping with their familial background were retrospectively ascertained through the database of the North West Genomic Laboratory Hub, Manchester, UK. The children were self-reported as unrelated. Only individuals for whom a referral for genetic testing was received between June 2015 and June 2018 (36 months total) were included.

All study participants were examined in tertiary paediatric ophthalmic genetic clinics at Manchester University Hospitals, Manchester, UK. A 3-generation pedigree and full ocular, developmental and medical history were obtained for each patient; this included questioning about skin and hair pigmentation at birth, ability to tan, and ease of burning on sun exposure; eye colour was also noted. Clinical assessment included visual acuity testing using age-appropriate optotypes, dilated fundus examination, cycloplegic refraction and orthoptic assessment; the dermatological phenotype at the time of consultation was also recorded.

Optical coherence tomography (OCT) imaging was offered to children aged 4 or more years. The Spectralis HRA + OCT system (Heidelberg Engineering, Heidelberg, Germany) or the Topcon 3D OCT 2000 (Topcon GB, Newberry, Berkshire, UK) were used. Due to limited compliance and the presence of nystagmus it was possible to obtain reliable scans of the fovea in only 6/12 study subjects.

Electrodiagnostic testing to look for optic nerve misrouting was performed in 10/12 cases. The protocols used incorporated the standards of the International Society for Electrophysiology of Vision (ISCEV) for clinical VEPs^41,42^; both pattern onset (30’ checks, 200 ms on, 800 ms off) and flash (Grass #4, 1 Hz) stimulation were attempted in all tested subjects. The tests were carried out using either an Espion2 or Espion3 clinical electrophysiology system (Diagnosys LLC, Lowell, MA, USA), with flash stimulation provided by a PS33 photic stimulator (Grass Technologies, Warwick, RI, USA). Misrouting was assessed using an index of crossed asymmetry, looking at the difference between the VEP recorded 3 cm to the right and left of the occipital midline and comparing right and left eye stimulation. Results were graded using the following scale: −1: No response recordable; 0: No crossed asymmetry (right-left scalp difference plots were the same for the right and left eye); +1: Possible crossed asymmetry (wherein most VEP components did not cross, but minor components did); +2: Probable crossed asymmetry (partial crossed asymmetry, where asymmetry is only seen in one eye, or the polarity of the difference plots is opposite but partially out of phase); +3: Definite crossed asymmetry (difference plots are mirror images of each other). An alternative approach is to use the asymmetry index^43^. However, whereas the asymmetry index utilises a single time slice, our method considers the whole range of the visual response. Notably, we have opted to use this subjective grading technique as opposed to other, more objective approaches that evaluate the whole response (such as Pearson’s correlate^44^ or the chiasmal coefficient^45^) for two reasons. Firstly, both Pearson’s correlate^44^ and the chiasmal coefficient^45^ do not take data scale into consideration. As a result, when there is no signal, background noise can give an artificially high numeric measure of crossed asymmetry. Secondly, significant but localised optic nerve misrouting may be masked by high positive correlation of the rest of the waveform. In any case, our approach was compared to both Pearson’s correlate and chiasmal coefficient and we found these three methods to be in good agreement.

### Clinical genetic testing and bioinformatic analysis

Gene panel testing and analysis were performed at the North West Genomic Laboratory Hub Genomic Diagnostic Laboratory, a UK Accreditation Service Clinical Pathology Accredited medical laboratory (Clinical Pathology Accredited identifier, no. 4015). DNA was extracted from peripheral blood samples and subsequently processed using Agilent SureSelect target-enrichment kits (Agilent, Santa Clara, CA, USA). These were designed to capture all exons and 50 base pairs of flanking introns of 193 genes associated with inherited ophthalmic disease. Analysis was then limited to a panel of clinically relevant genes, chosen by the referring clinician. For our selected partial albinism cases, the panels used for analysis included: an OA/OCA panel consisting of 18 genes (5/12 cases), a nystagmus & foveal hypoplasia panel consisting of 26 genes (encompassing the genes included in the OA/OCA panel; 5/12 cases), an optic nerve disorders panel consisting of 40 genes (encompassing both the OA/OCA and the nystagmus & foveal hypoplasia panel genes; 1/12); a list of all tested transcripts and genes can be found in Supplementary Table S1. One study participant (proband 9) underwent clinical exome sequencing using a specifically designed Agilent SureSelect target-enrichment kit.

A detailed description of our sequencing pipeline and downstream bioinformatics analysis has been previously reported^46^. Briefly, after enrichment, the samples were sequenced on an Illumina HiSeq. 2000/2500 system (Illumina, Inc, San Diego, CA) according to the manufacturer’s protocols. Sequence reads were subsequently demultiplexed using CASAVA software version 1.8.2 (Illumina, Inc, San Diego, CA) and aligned to the hg19 reference genome using the Burrows Wheeler Aligner (BWA-short version 0.62)^47^. Duplicate reads were removed using Samtools before base quality score recalibration and insertion-deletion realignment using the Genome Analysis Tool Kit (GATK-lite version 2.0.39)^48^. The UnifiedGenotyper within the Genome Analysis Tool Kit was used for single nucleotide variant and insertion-deletion discovery^49^. To reduce the number of false-positive variants, we primarily limited the clinical analysis to changes with sequencing quality metrics above specific criteria: single nucleotide variants with ≥50x independent sequencing reads and ≥45 mean quality value, and insertions-deletions with support from >25% of the aligned and independent sequencing reads were considered^46^. Copy number variants were detected from high-throughput sequencing read data using ExomeDepth version 1.1.6^46,50^.

Clinical interpretation of variants was broadly consistent with the 2015 American College of Medical Genetics and Genomics best practice guidelines^51^. The Ensembl Variant Effect Predictor^52^ was used to predict functional consequences of the identified genetic changes, and a pathogenicity classification score^51^ was assigned after extensive appraisal of the scientific literature, and the patient’s clinical referral. In silico modelling was also used, utilising PolyPhen-2^53^ and Combined Annotation-Dependent Depletion (CADD)^54^. A clinical report was then generated and variants that possibly or probably accounted for the tested individual’s clinical presentation were highlighted. Where possible, the samples of relatives were sought to identify zygosity and confirm pathogenicity.

For the purpose of this study, participants were split into 3 groups, as previously described in Taylor *et al*.^55^:

- Probable molecular diagnosis group: patients with clearly or likely disease-associated variant(s) in an apparently disease-causing state (e.g., ≥1 variant in a gene linked with dominant disease or ≥2 variants in a gene linked with recessive disease).
- Possible molecular diagnosis group: cases harboring a single clearly or likely disease-associated variant in a gene linked with recessive disease, provided the patient phenotype matches the known spectrum of clinical features for this gene; it can be speculated that most of these participants will carry a second variant that could not be detected.
- Unknown molecular diagnosis group: all other cases for which no clearly or likely disease-associated variants were detected.

### Evaluating the prevalence of selected genotypes in an external cohort

After identifying some unexpected combinations of *TYR* variants in the patient cohort, we aimed to ascertain how frequently these combinations were encountered in individuals without a clinical diagnosis of albinism. We interrogated genomic data from 4046 individuals within the 100,000 genomes pilot dataset^25^. None of these individuals had features suggestive of albinism. The data were accessed via remote desktop after the relevant participant agreement was signed. The following questions were asked:

- How many of the 4046 individuals have the *TYR* p.[(Ser192Tyr);(Arg402Gln)] allele in homozygous state?
- How many of the 4046 individuals have the *TYR* p.[(Ser192Tyr);(Arg402Gln)] allele in heterozygous state AND another rare *TYR* variant?
- How many of the 4046 individuals have a rare *TYR* variant AND a rare *OCA2* variant?

To define rare for the purposes of this experiment, we interrogated the gnomAD variant database^14^ (accessed 23/01/2019) for known disease-associated variants in both *OCA2* and *TYR*. The minor allele frequency (MAF) of the most prevalent *OCA2* or *TYR* mutation was used as cut-off: this corresponded to 0.047, the frequency of the *OCA2* c.1441 G > A, (p.Ala481Thr) variant in the gnomAD European Finnish population.

### Ethics approval

Ethics approval was obtained from the North West Research Ethics Committee (11/NW/0421 and 15/YH/0365).

### Provenance and peer review

Externally peer reviewed.

## Supplementary information

Supplementary material for "clinical and genetic variability in children with partial albinism"
