# Supplementary material for: Clinical and genetic variability in children with partial albinism
Source: Sci Rep. 2019 Nov 12;9:16576. doi: 10.1038/s41598-019-51768-8 (PMC6851142; doi:10.1038/s41598-019-51768-8)
Supplement: Supplementary file 1 — Supplementary material for "clinical and genetic variability in children with partial albinism" [file 41598_2019_51768_MOESM1_ESM.pdf]

ONLINE SUPPLEMENTARY MATERIAL

Clinical and genetic variability in children with partial albinism

Patrick Campbell [1†], Jamie M. Ellingford [1,2†], Neil R. A. Parry [3], Tracy Fletcher [1], Simon C. Ramsden [1], Theodora Gale [1], Georgina Hall [1], Katherine Smith [4], Dalia Kasperaviciute [4], Ellen Thomas [4], I. Chris Lloyd [1,5], Sofia Douzgou [1,2], Jill Clayton-Smith [1,2], Susmito Biswas [3], Jane L. Ashworth [3], Graeme C. M. Black [1,2], Panagiotis I. Sergouniotis [1,2,3]

[1] Manchester Centre for Genomic Medicine, Manchester Academic Health Sciences Centre, Manchester University NHS Foundation Trust, St Mary's Hospital, Manchester, UK.

[2] Division of Evolution and Genomic Sciences, Faculty of Biology, Medicine and Health, University of Manchester, Manchester, UK,

[3] Manchester Royal Eye Hospital, Manchester Academic Health Science Centre, Manchester University NHS Foundation Trust, Manchester, UK

[4] Genomics England, London, UK

[5] Great Ormond Street Hospital for Children NHS Foundation Trust, London, UK

[†]PC and JE are joint first authors

Supplementary Table 1: Gene panels used in this study

Optic nerve disorders panel (40 genes; including foveal hypoplasia & ocular/oculocutaneous albinism panels)

Gene (HGNC)	Transcript
ACO2	NM_001098.2
AP3B1	NM_003664.4
ATOH7	NM_145178.3
BLOC1S3	NM_212550.4
C10orf11	NM_032024.3; NM_001305581.1
C12orf65	NM_152269.4
CACNA1F	NM_005183.3; NM_001256790.2
CISD2	NM_001008388.4
DHCR7	NM_001360.2
DTNBP1	NM_032122.4; NM_183040.2
FRMD7	NM_194277.2
GJA1	NM_000165.3
GPR143	NM_000273.2
HMX1	NM_018942.2
HPS3	NM_032383.4
HPS4	NM_022081.5; NM_152841.2
HPS5	NM_181507.1
HPS6	NM_024747.5
LARGE	NM_004737.4
LYST	NM_000081.3
OCA2	NM_000275.2
OPA1	NM_015560.2; NM_130837.2
OPA3	NM_025136.3; NM_001017989.2
PAX6	NM_001604.4
PLDN (BLOC1S6)	NM_001311255.1; NM_012388.3
RAB18	NM_021252.3
RAB3GAP1	NM_001172435.1
RAB3GAP2	NM_012414.3
RTN4IP1	NM_032730.4
SIX6	NM_007374.2
SLC24A5	NM_205850.2
SLC38A8	NM_001080442.1
SLC45A2	NM_016180.4; NM_001012509.3
SLC4A11	NM_032034; NM_001174089.1; NM_001174090.1
SOX2	NM_003106.3
TBC1D20	NM_144628.3
TMEM126A	NM_032273.3
TYR	NM_000372.4
TYRP1	NM_000550.2
WFS1	NM_006005.3

Foveal hypoplasia & nystagmus panel (26 genes; including ocular/oculocutaneous albinism panel)

Gene (HGNC)	Transcript
ACO2	NM_001098.2
AP3B1	NM_003664.4
ATOH7	NM_145178.3
BLOC1S3	NM_212550.4
C10orf11	NM_032024.3; NM_001305581.1
CACNA1F	NM_005183.3; NM_001256790.2
DTNBP1	NM_032122.4; NM_183040.2
FRMD7	NM_194277.2
GPR143	NM_000273.2
HMX1	NM_018942.2
HPS3	NM_032383.4
HPS4	NM_022081.5; NM_152841.2
HPS5	NM_181507.1
HPS6	NM_024747.5
LYST	NM_000081.3
OCA2	NM_000275.2
PAX6	NM_001604.4
PLDN (BLOC1S6)	NM_001311255.1; NM_012388.3
RTN4IP1	NM_032730.4
SIX6	NM_007374.2
SLC24A5	NM_205850.2
SLC38A8	NM_001080442.1
SLC45A2	NM_016180.4; NM_001012509.3
SLC4A11	NM_032034; NM_001174089.1; NM_001174090.1
TYR	NM_000372.4
TYRP1	NM_000550.2

Ocular/oculocutaneous albinism panel (18 genes)

Gene (HGNC)	Transcript
AP3B1	NM_003664.4
BLOC1S3	NM_212550.4
C10orf11	NM_032024.3; NM_001305581.1
CACNA1F	NM_005183.3; NM_001256790.2
DTNBP1	NM_032122.4; NM_183040.2
GPR143	NM_000273.2
HPS3	NM_032383.4
HPS4	NM_022081.5; NM_152841.2
HPS5	NM_181507.1
HPS6	NM_024747.5
LYST	NM_000081.3
OCA2	NM_000275.2
PLDN (BLOC1S6)	NM_001311255.1; NM_012388.3
SLC24A5	NM_205850.2
SLC45A2	NM_016180.4; NM_001012509.3
SLC4A11	NM_032034; NM_001174089.1; NM_001174090.1
TYR	NM_000372.4
TYRP1	NM_000550.2

It was not possible to gain acceptable coverage of *HPS1* and it was therefore not included in these clinical diagnostic panels.
AP3D150, *MITF51*, and *GNAI3* were also not included.

Supplementary File: Family trees of children with partial albinism

Proband 1

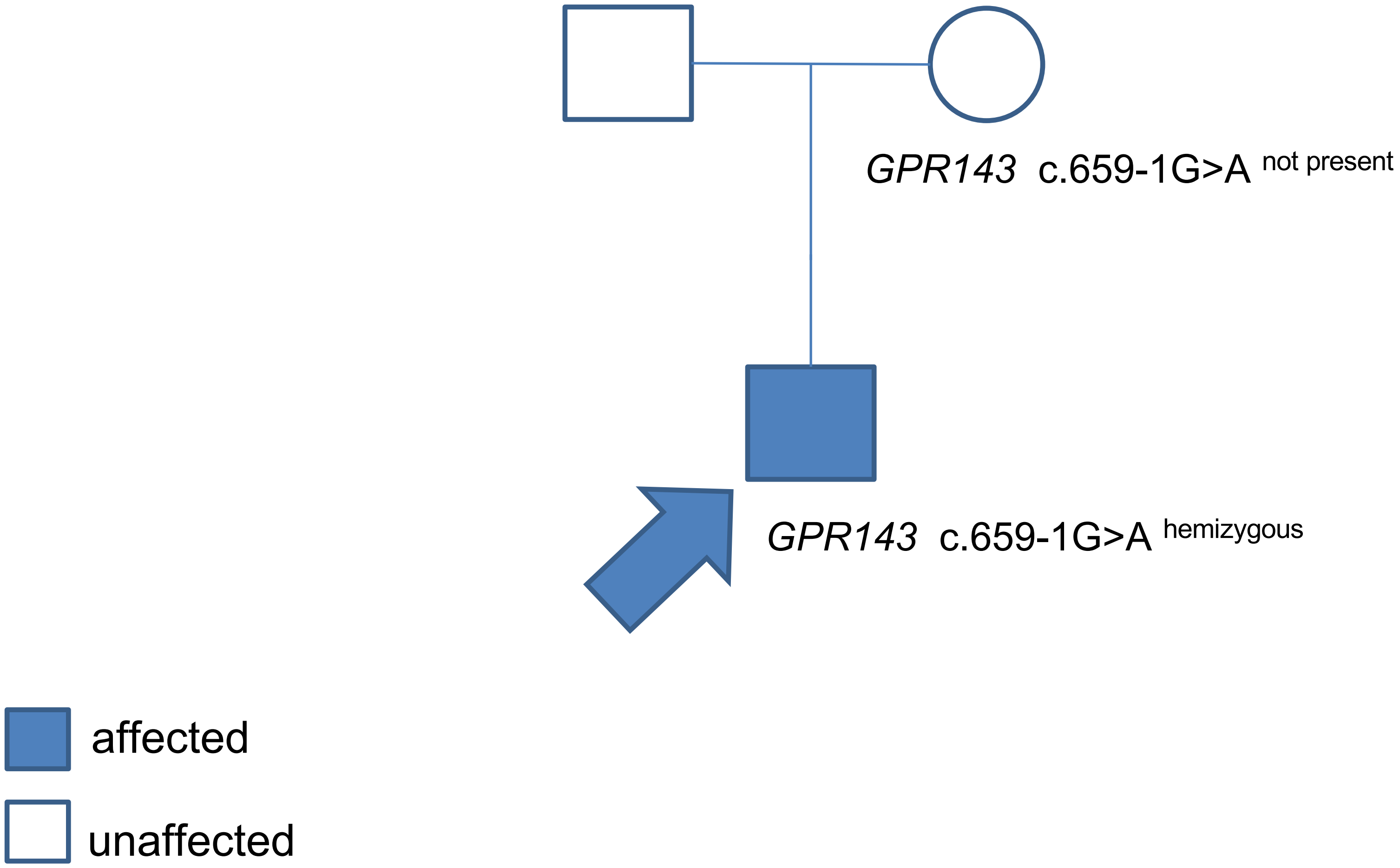

Proband 4

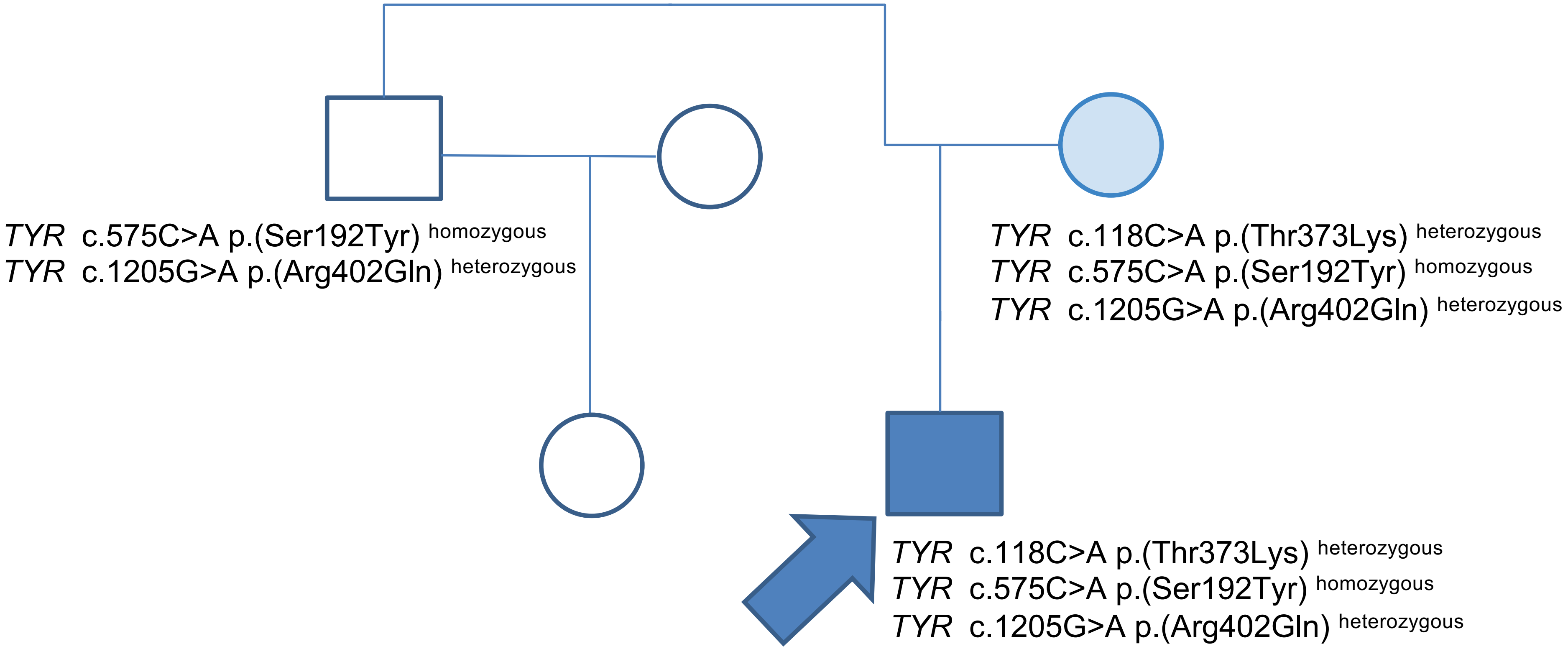

intermediate phenotype
(mild transillumination, foveal
hypoplasia but no nystagmus)

Proband 5

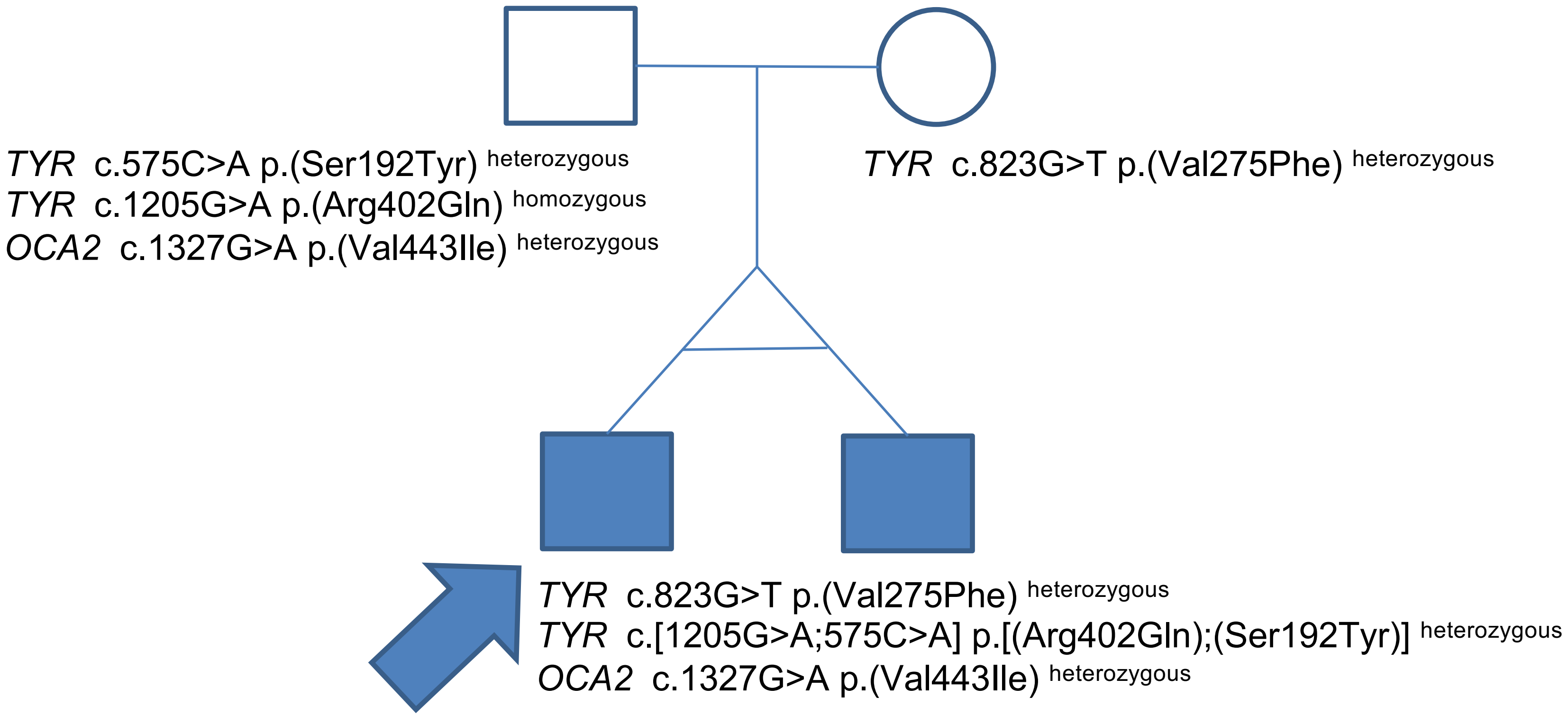
TYR c.575C>A p.(Ser192Tyr) heterozygous
TYR c.1205G>A p.(Arg402Gln) homozygous
OCA2 c.1327G>A p.(Val443Ile) heterozygous
TYR c.823G>T p.(Val275Phe) heterozygous
TYR c.823G>T p.(Val275Phe) heterozygous
TYR c.[1205G>A;575C>A] p.[(Arg402Gln);(Ser192Tyr)] heterozygous
OCA2 c.1327G>A p.(Val443Ile) heterozygous

Proband 6

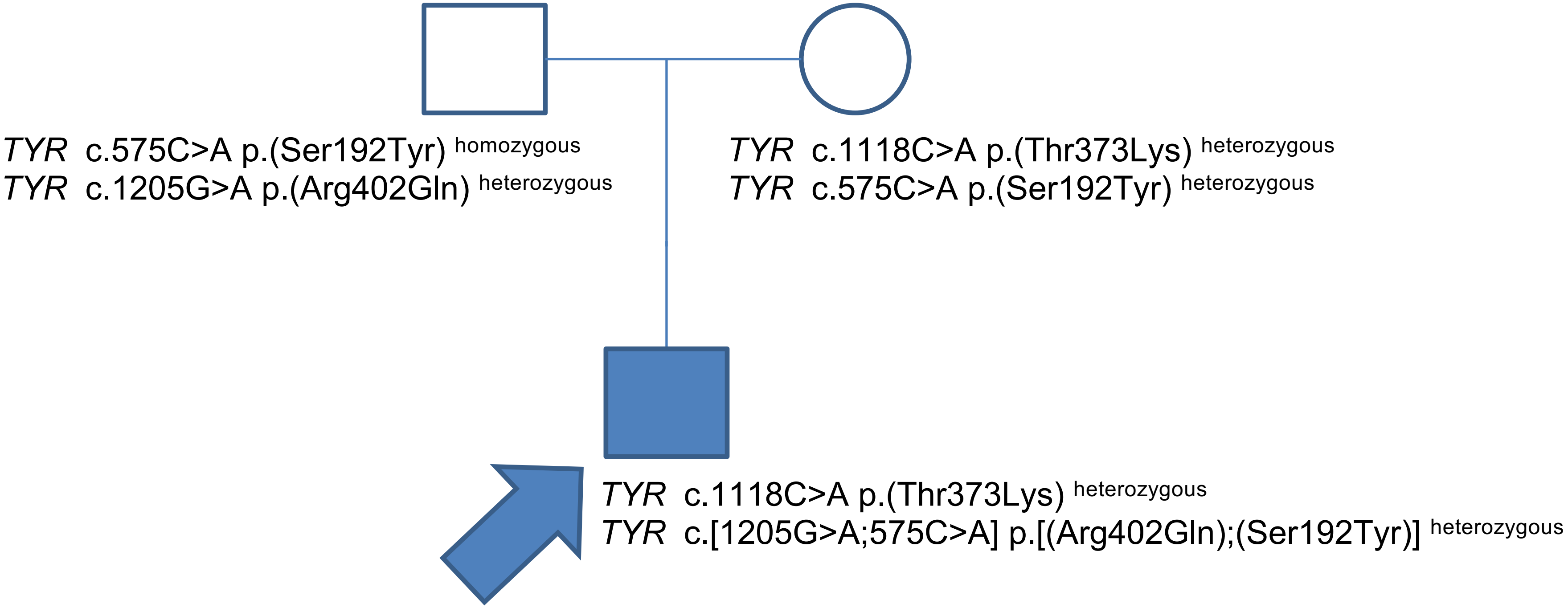
TYR c.575C>A p.(Ser192Tyr) homozygous
TYR c.1205G>A p.(Arg402Gln) heterozygous
TYR c.1118C>A p.(Thr373Lys) heterozygous
TYR c.575C>A p.(Ser192Tyr) heterozygous
TYR c.1118C>A p.(Thr373Lys) heterozygous
TYR c.[1205G>A;575C>A] p.[(Arg402Gln);(Ser192Tyr)] heterozygous

Proband 8

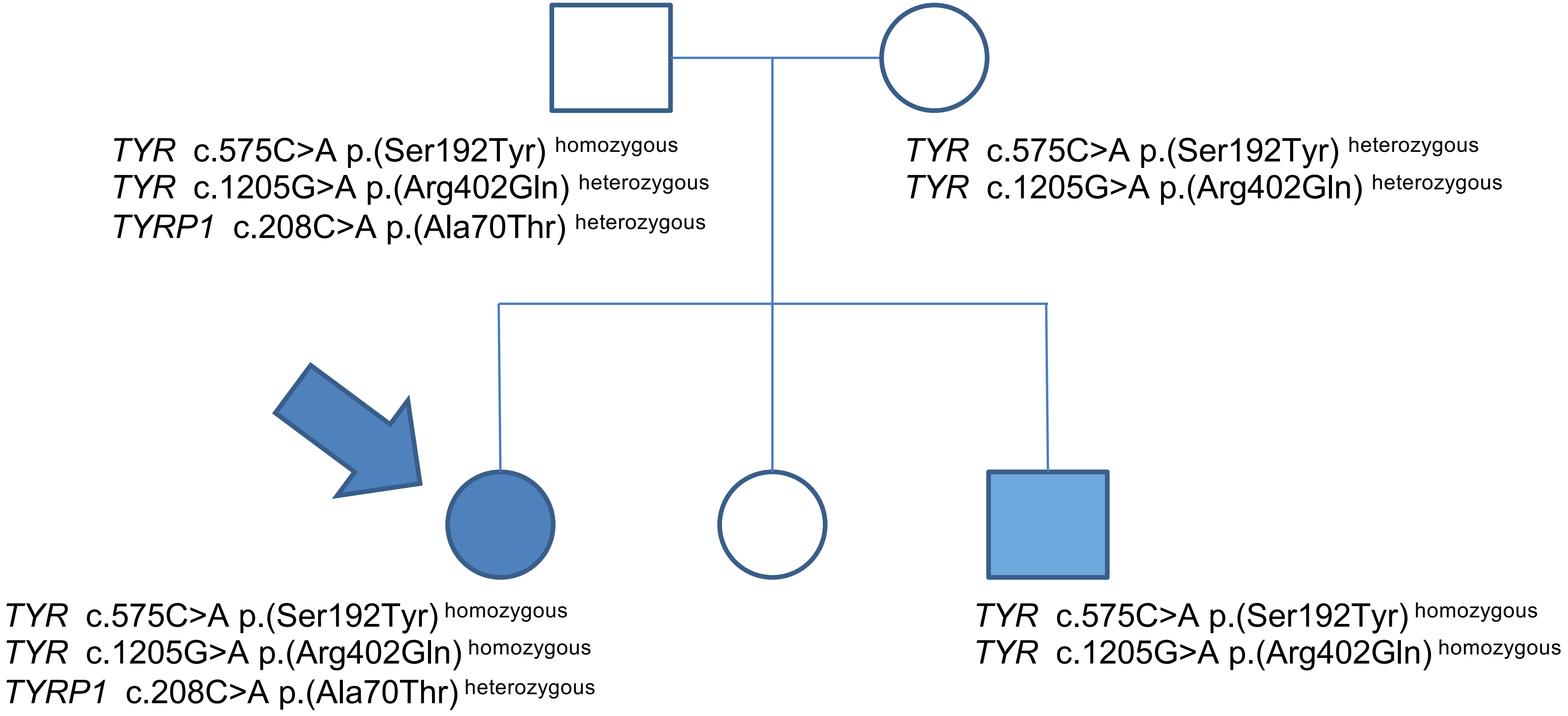

Likely ocular albinism phenotype (nystagmus present but no clinical tests performed yet as <1y old)

Proband 10

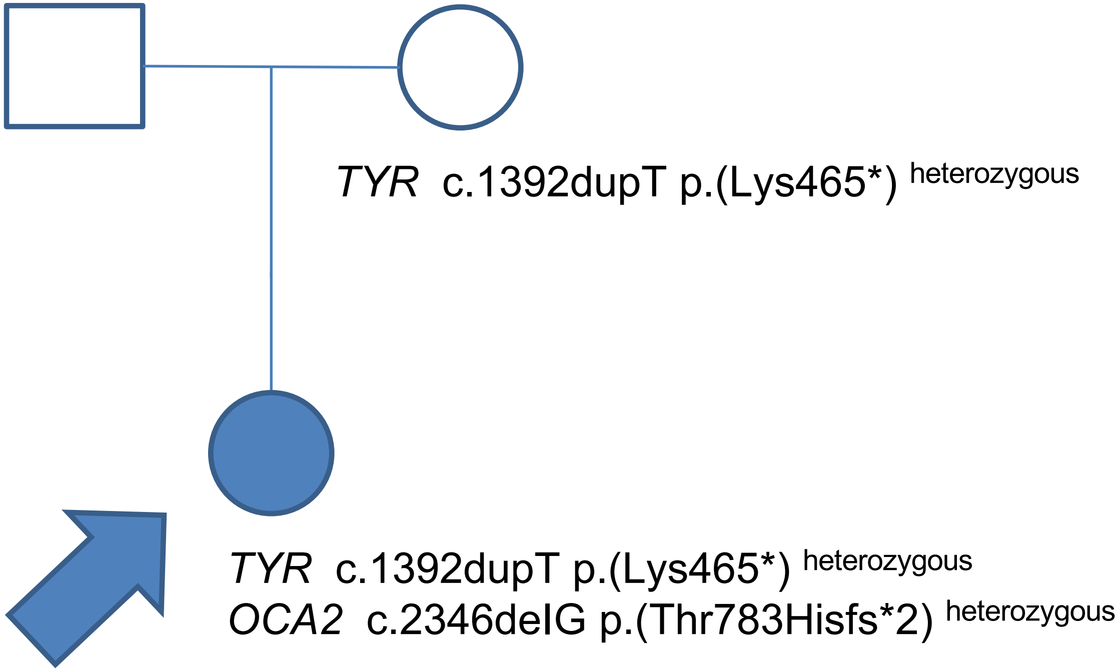
TYR c.1392dupT p.(Lys465*) heterozygous
TYR c.1392dupT p.(Lys465*) heterozygous
OCA2 c.2346delG p.(Thr783Hisfs*2) heterozygous

Proband 11

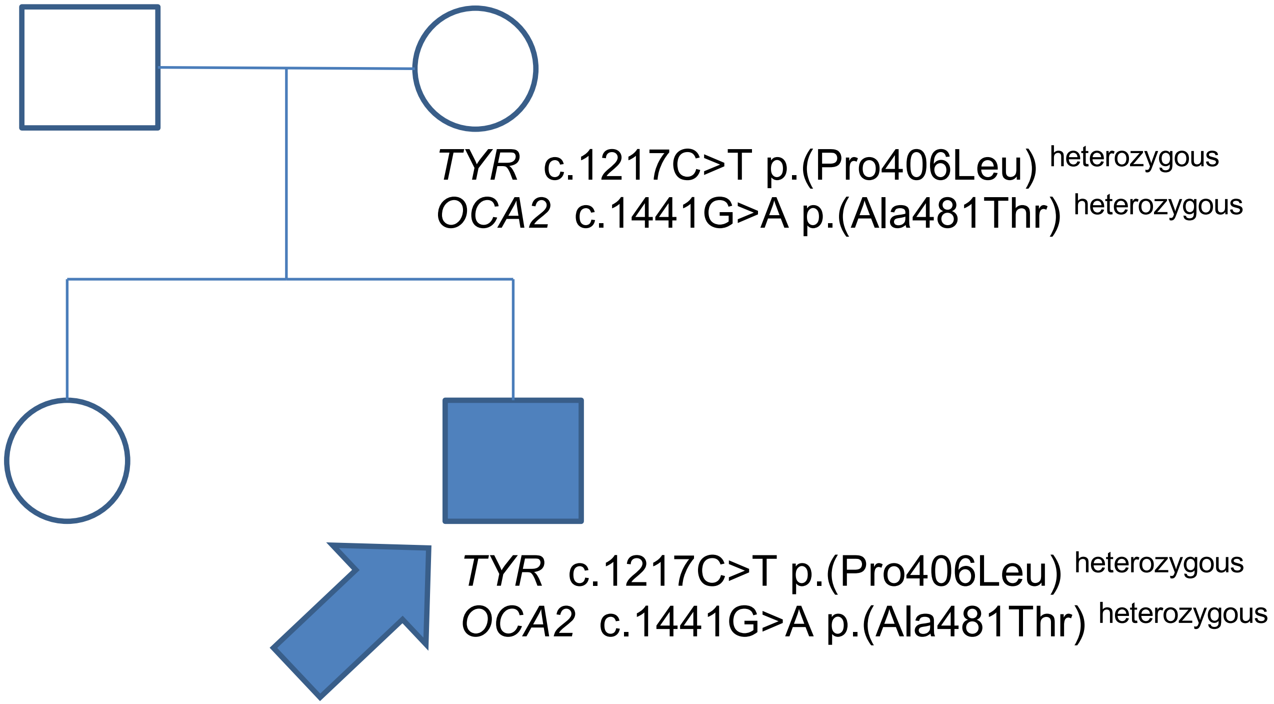
TYR c.1217C>T p.(Pro406Leu) heterozygous
OCA2 c.1441G>A p.(Ala481Thr) heterozygous
TYR c.1217C>T p.(Pro406Leu) heterozygous
OCA2 c.1441G>A p.(Ala481Thr) heterozygous
